# Differences in risk of serious infections between patients with secondary versus primary nephropathy following rituximab treatment: a retrospective cohort study

**DOI:** 10.3389/fimmu.2024.1390997

**Published:** 2024-06-11

**Authors:** Jing Xu, Ying Ding, Zhen Qu, Feng Yu

**Affiliations:** Department of Nephrology, Peking University International Hospital, Beijing, China

**Keywords:** autoimmune kidney disease, rituximab, severe infection, secondary nephropathy, risk factors

## Abstract

**Background:**

The incidence of severe infections (SIs) in patients with autoimmune nephropathy after rituximab (RTX) treatment varies significantly. Our study aims to identify high-risk populations, specifically by comparing the differences in the risk of SIs between patients with primary nephropathy and those with nephropathy in the context of systemic autoimmune diseases (referred to as secondary nephropathy).

**Methods:**

This retrospective cohort study investigated the occurrence of SIs in adult patients with immune-related kidney disease who received RTX treatment at our institution from 2017 to 2022. Multivariable COX regression models were used to analyze the association between the type of nephropathy (primary or secondary) and SIs. Propensity score analyses, subgroup analyses, and E-value calculations were performed to ensure the reliability of the results.

**Results:**

Out of 123 patients, 32 (26%) developed 39 cases of SIs during a mean follow-up period of 19.7 ± 14.6 months post-RTX treatment, resulting in an incidence rate of 18.9/100 patient-years. The multivariable COX regression analysis indicated that patients with secondary nephropathy had a significantly higher risk of SIs compared to those with primary nephropathy (HR = 5.86, 95% CI: 1.05–32.63, P = 0.044), even after accounting for confounding variables including gender, age, BMI, history of prior SIs, baseline eGFR, lymphocyte counts, IgG levels, and the utilization of other immunosuppressive therapies. Various sensitivity analyses consistently supported these findings, with an E-value of 5.99. Furthermore, advanced age (HR: 1.03; 95% CI: 1.01–1.06; P = 0.023), low baseline IgG levels (HR: 0.75; 95% CI: 0.64–0.89; P < 0.001), and recent history of SIs (HR: 5.68; 95% CI: 2.2–14.66; P < 0.001) were identified as independent risk factors.

**Conclusion:**

The incidence of SIs following RTX administration in patients with autoimmune nephropathy is significant. It is crucial to note that there are distinct differences between the subgroups of primary and secondary nephropathy. Patients with secondary nephropathy, particularly those who are elderly, have low baseline IgG levels, and have a recent history of SI, are more susceptible to SIs.

## 1 Introduction

Recently, rituximab (RTX) has gained popularity as an immunosuppressant in the treatment of autoimmune kidney disease. The safety of RTX has become a growing concern, particularly due to severe infections (SIs) which can lead to significant morbidity and mortality in patients with kidney disease.

According to the literature, the incidence of SIs following RTX treatment in patients with renal disease varies significantly depending on the disease. Categorizing patients based on primary nephropathy and those with nephropathy in the context of systemic autoimmune diseases (referred to as secondary nephropathy) highlights a distinct pattern. In primary nephropathy (PN), encompassing membranous nephropathy (MN), microscopic lesion disease (MCD), and focal segmental glomerulosclerosis (FSGS), the incidence of SIs is generally low, often reported as 0 in multiple studies, and typically below 6% (1–6). Conversely, patients with secondary nephropathies (SN) such as lupus nephritis (LN) and antineutrophil cytoplasmic antibody-associated vasculitis (AAV) exhibit a higher prevalence of SIs, ranging from 15.8% to 19.2% (7, 8) and 18% to 38% (9–11), respectively. This discrepancy has sparked significant interest. Are SIs following RTX therapy associated with the type of nephropathy? Do patients with SN face a higher infection risk compared to those with PN?

To the best of our knowledge, only two studies have specifically investigated the incidence and risk factors of SIs following RTX treatment in the unique population of patients with primary autoimmune kidney diseases. Trivin et al. conducted a study in France involving 98 patients diagnosed with membranoproliferative glomerulonephritis (MPGN), MCD, LN, and AAV, and found a SI incidence rate of 21.6/100 person-years within a 12.7-month follow-up period after RTX administration (12). Another study by Odler et al. in Austria included 83 patients with nephritis (primarily AAV) and 61 patients with kidney disease (primarily MN). During an average follow-up period of 2.2 (0–4.9) years, 17.4% of patients experienced SIs within 1 year after RTX treatment (13). Both of these studies indicated a higher overall incidence of SIs in patients with primary autoimmune kidney diseases following RTX therapy. However, the screening results for risk factors differed. Trivin et al. identified diabetes, cumulative RTX dose, and concomitant use of azathioprine as independent risk factors for SIs (12), while Odler et al. found that body mass index (BMI) and baseline creatinine levels were significantly associated with SIs (13). Notably, these studies are from European countries, with limited data from Asian populations, and did not address the aforementioned questions of interest. Although Odler et al. observed a seemingly higher incidence of SIs in the nephritis group compared to the nephropathy group following RTX treatment (without statistical significance) (13), the grouping based primarily on urinary protein quantification levels may not effectively distinguish between primary and secondary nephropathy, as the latter is based on the presence of systemic autoimmune diseases in patients. Therefore, it remains uncertain whether there are differences in infection risk between patients with primary and secondary nephropathy.

Our study aimed to investigate the incidence of SIs in renal patients treated with RTX in our center (Asian population cohort) and to screen for risk factors. Specifically, we compared the risk of infection in patients with SN to those with PN.

## 2 Methods

### 2.1 Study design, population, and setting

This is a retrospective cohort study of patients at the Department of Nephrology of Peking University International Hospital. Patients with immune-related kidney diseases receiving RTX between March 1, 2017 and December 31, 2022 were included. Additional inclusion criteria consisted of age ≥18 years at the start of RTX therapy and treated with at least one dose of RTX. Follow-up was until May 1, 2023. Person-time in the study spanned between the patients’ first RTX dose and their last follow-up date or the end of the study period, whichever occurred first. Exclusion criteria were defined as follows: (1) patients with concomitant malignancy and (2) follow-up time <1 month.

### 2.2 Data collection

All clinical and laboratory data were collected from available medical records of the patients. We collected data regarding patient clinical characteristics, comorbidities, previous immunosuppressant use, cumulative dose of glucocorticoids (GCs), history of SIs, dialysis treatment, detailed RTX administration regimen, medication combination, and prophylactic anti-infective drugs. Laboratory results were collected at baseline (before RTX infusion) and at 3, 6 and 12 months after treatment initiation and every 12 months until the last follow-up.

The cumulative dose of GCs was determined by calculating the total amount of prednisone or prednisone equivalent administered within 3 months from RTX administration. Dialysis was defined as hemodialysis or peritoneal dialysis initiated prior to RTX administration. History of serious infection was defined as any serious infection that occurred within 3 months prior to RTX administration. The estimated glomerular filtration rate (eGFR) was calculated by the Chronic Kidney Disease Epidemiology Collaboration equation.

### 2.3 Prophylactic medicine

In our center, all patients with kidney disease who are treated with RTX receive infection prevention based on the same criteria. The criteria for prophylactic use of TMP-SMX are as follows: (1) CD4+ T-lymphocyte count below 200 cells/microliter or less than 20% of total circulating lymphocytes, or a previous episode of Pneumocistis Jyrovecii (Carinii) pneumonia based on the Recommendations for Prevention of Pneumocystis carinii Pneumonia for Adults and Adolescents Infected with Human Immunodeficiency Virus, published by the Centers for Disease Control (MMWR Recommendations and Reports, 1992;41(RR-4):1–11). (2) For patients with AAV, prophylactic TMP-SMX is routinely administered during immunosuppressive therapy after excluding contraindications to dosing. This criterion is based on the KDIGO 2021 Clinical Practice Guidelines for the Management of Glomerular Disease (Kidney Disease: Improving Global Outcomes Glomerular Diseases Work Group, Kidney Int. 2021;100(4S): S198). Entecavir prophylaxis is indicated for either of the following two criteria: (1) HBsAg-positive HBV carriers, and (2) HBsAg-negative, HBcAb-positive patients. Isoniazid is administered as prophylaxis to patients with latent tuberculosis infection, especially those with a strongly positive PPD test. Ganciclovir is used as prophylactic antiviral therapy for patients with CMV viremia, indicated by a serum CMV-DNA level greater than 0.

### 2.4 Primary outcome

SIs were defined as infections that required hospitalization and/or intravenous anti-infective therapy and/or resulted in death. Infections were graded according to the Common Terminology Criteria for Adverse Events (CTCAE) version 5.0 (14). Clinical and hospitalization reports were reviewed during RTX treatment to identify severe (grade 3–5) infections. The site of infections was categorized into the upper respiratory tract (nose, paranasal sinuses, pharynx, larynx, or trachea), lower respiratory tract (trachea and lungs), gastrointestinal tract, urinary tract, skin or soft tissue, and other infections. Pathogens were classified as bacteria, fungi, viruses, and tuberculosis. Pathogens were identified through various methods including smears, cultures, NGS tests, serum-specific antigen, antibody, or nucleic acid tests of body fluids or secretions. This identification was done in combination with other laboratory markers, imaging studies, and clinical manifestations. In cases where direct evidence related to pathogenesis was lacking, a consultative opinion from a physician with the appropriate specialty was sought. Especially, for patients with respiratory tract infections due to bacteria, we determined its presence by conducting smear, culture, and NGS testing on sputum or alveolar lavage samples. Additionally, we considered blood counts, CRP, and PCT test results, as well as imaging changes in the lungs and the patient’s clinical presentation. And if needed, we sought consultative opinions from respiratory specialists. The primary outcome of this study was the occurrence of SIs during the follow-up period after the first RTX administration.

### 2.5 Statistical methods

Categorical variables were expressed as proportions (%). Continuous data were expressed as mean and standard deviation or median and interquartile range (IQR), as appropriate. Baseline characteristics and investigated possible risk factors for SIs at the start of RTX therapy are presented for all patients and for patients with PN and patients with SN. For continuous variables with less than 10% missing data at baseline, we used the mean or median of the variable for replacement. However, variables with more than 10% missing data were deleted. One way analysis of variance (normal distribution), Kruskal–Wallis H (skewed distribution) test, Chi-square tests or Fisher’s exact tests (categorical variables) were used to determine any statistical differences between the means and proportions of the groups. Univariable and multivariable Cox regression analyses were performed to test the association between candidate risk factors and SIs. Time to event was defined as time from the start of RTX to SIs or death, loss to follow-up or the end of the study period, whichever occurs first. Kaplan–Meier analysis was used to compare long-term survival rates in patients without SIs across different subgroups according to their type of nephropathy.

In the multivariable analysis, addressing confounding factors is crucial. We employed various statistical models to ensure the stability of our results. In the final model, we adjusted the factors based on three key rules (1 or 2 or 3) (15–17): (1) Variables were adjusted if their inclusion in the model resulted in a change of at least 10 percent in the matched odds ratio. (2) For univariate analysis, variables with p-values less than 0.05 were adjusted. (3) In the multivariable analysis, variables were selected based on previous research, clinical knowledge, and constraints. Therefore, the results from the final model (Model III) are more robust and conservative compared to other models. The detailed selection criteria for factors in each model are outlined as follows: Factors were chosen when added it to this model, changed the matched odds ratio by at least 10 percent in model I. Factors were chosen when in univariate analysis, their p values were less than 0.05 or when added it to this model, changed the matched odds ratio by at least 10 percent in model II. Factors meeting the aforementioned criteria or aligning with previous research, clinical expertise and constraints were chosen in model III.

### 2.6 Sensitivity analyses

We employed a range of propensity score (PS) analyses to evaluate the relationship between nephropathy type and SIs. These analyses included propensity score-matched analysis (PSM), utilization of PS as a covariate, inverse probability of treatment weighting (IPW), standardized mortality ratio weighting (SMRW), pairwise algorithmic (PA), and overlap weight (OW) regression analysis (18). The propensity score was calculated by using logistic regression with subgroup as outcome and including sex, age, BMI, disease duration, RTX treatment indication (relapse or refractory/Initial treated disease), cumulative dose of RTX, co-use of GCs, co-use of immunosuppressant, and baseline IgG levels as covariates. Standardized mean difference (SMD) was calculated to evaluate the efficiency of PSM in reducing the differences between the two groups. Less than 0.1 was considered an acceptable threshold. To address potential selection bias, subgroup analyses were performed separately after excluding patients with missing IgG levels at baseline and excluding patients who were on dialysis prior to RTX treatment. Furthermore, the study evaluated the possibility of unmeasured confounding between nephropathy subgroups and SIs by calculating the E-value (19).

All analyses were performed using the statistical software packages R (http://www.R-project.org, The R Foundation) and Free Statistics software versions 1.8. A two-tailed test was performed and P < 0.05 was considered statistically significant.

## 3 Results

### 3.1 Population

**Figure 1:**
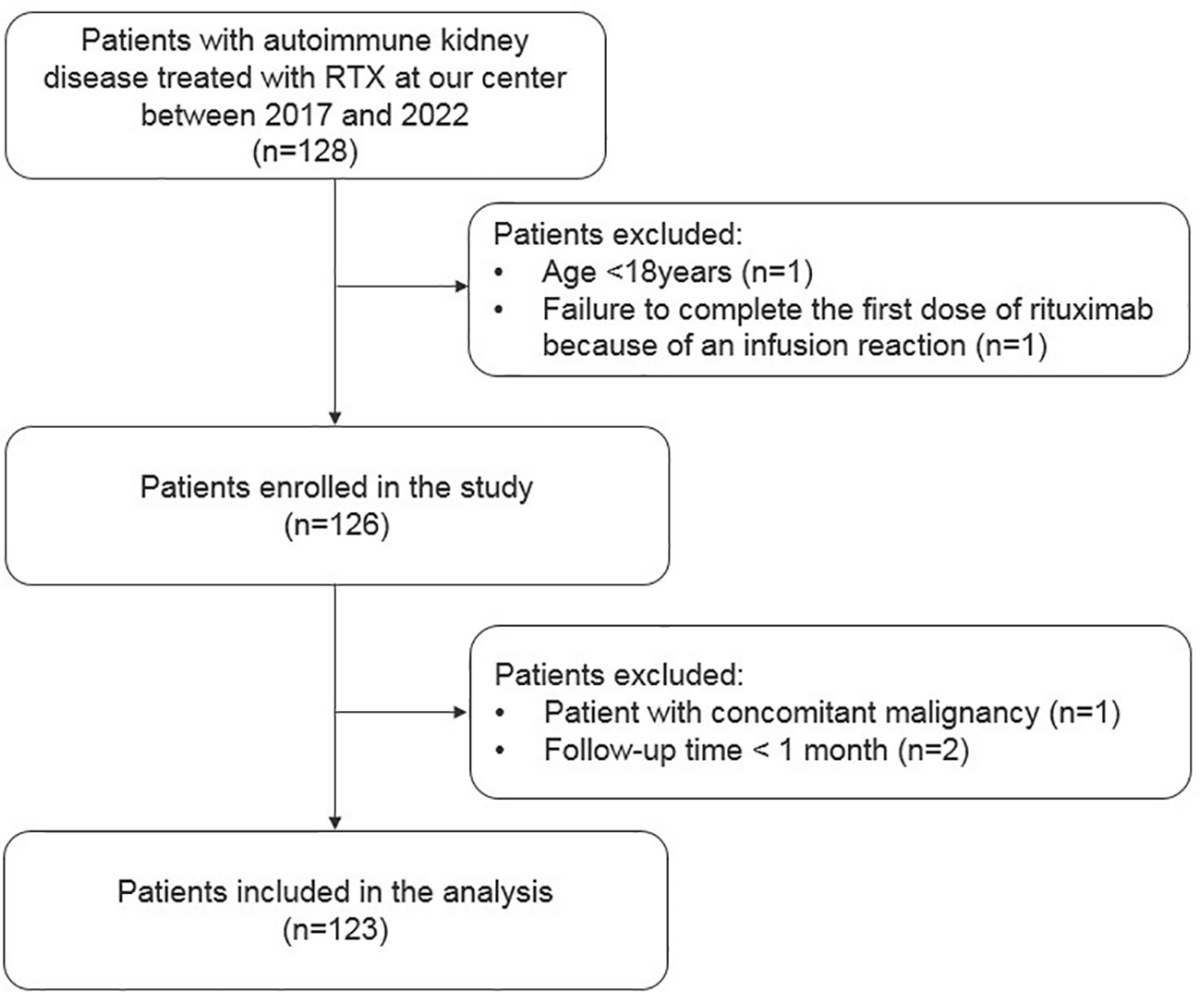
Flowchart of the study cohort.

Overall, 123 patients with autoimmune kidney diseases were included; 56 had primary glomerular disease and belonged to the PN group, whereas 67 patients had kidney involvement due to systemic autoimmune disorders and belonged to the SN group. The flow chart illustrating the inclusion and exclusion of patients is shown in **Figure 1**. Most patients in SN group had LN, while MN was the leading diagnosis in the PN group (30% and 34%, respectively). Detailed baseline demographic and clinical characteristics are shown in **Table 1**.

**Table 1:** Baseline characteristics of 123 patients receiving rituximab treatment.

Characteristics	Total (n = 123)	PN (n = 56)	SN (n = 67)	*p* Value
Age (years)	49.2 ± 18.3	52.2 ± 16.9	46.6 ± 19.1	0.087
Male, n (%)	74 (60.2)	45 (80.4)	29 (43.3)	< 0.001
BMI (kg/m^2^)	24.1 ± 3.6	25.3 ± 4.0	23.0 ± 2.9	< 0.001
Diagnose, n (%)				< 0.001
LN	37 (30.1)	0 (0)	37 (55.2)	
AAV	17 (13.8)	0 (0)	17 (25.4)	
Others ^a^	13 (10.6)	0 (0)	13 (19.4)	
MN	42 (34.1)	42 (75)	0 (0)	
MCD	11 (8.9)	11 (19.6)	0 (0)	
FSGS	3 (2.4)	3 (5.4)	0 (0)	
Disease duration (months)	20 (6, 72)	18 (8, 41)	29 (5, 120)	0.144
Comorbidities, n (%)				
Diabetes mellitus	23 (18.7)	18 (32.1)	5 (7.5)	< 0.001
Hypertension	43 (35.0)	25 (44.6)	18 (26.9)	0.039
Chronic lung disease	12 (9.8)	5 (8.9)	7 (10.4)	0.777
Recent history of SI^b^, n (%)	15 (12.2)	3 (5.4)	12 (17.9)	0.034
Previous treatments immunosuppressant, n (%)	81 (65.9)	36 (64.3)	45 (67.2)	0.038
MMF	24 (19.5)	5 (8.9)	19 (28.4)	0.007
CSA	28 (22.8)	24 (42.9)	4 (6)	< 0.001
CYC	50 (40.7)	12 (21.4)	38 (56.7)	< 0.001
LF	17 (13.8)	3 (5.4)	14 (20.9)	0.013
TAC	14 (11.4)	9 (16.1)	5 (7.5)	0.134
GCs, n (%)	97 (78.9)	39 (69.6)	58 (86.6)	0.022
Cumulative dose of GCs^c^ (g)	1.2 (0.0, 2.1)	0.6 (0.0, 1.4)	1.8 (0.7, 2.9)	< 0.001
Dialysis, n (%)	17 (13.8)	0 (0)	17 (25.3)	< 0.001
Baseline laboratory data				
eGFR (mL/min/1.73 m^2^)	52.0 (16.9, 89.4)	78.2 (55.6, 107.4)	19.3 (12.8, 48.6)	< 0.001
ALB (g/L)	30.5 (23.5, 34.4)	26.6 (20.0, 30.6)	33.0 (29.1, 36.2)	< 0.001
UTP (g/24 hour)	4.3 (2.3, 9.0)	7.7 (3.4, 13.4)	3.5 (1.3, 5.6)	< 0.001
WBC (10^9^/L)	8.4 ± 3.2	9.2 ± 3.2	7.6 ± 3.0	0.004
Lymphocytes (10^9^/L)	1.5 ± 0.9	1.9 ± 0.9	1.2 ± 0.7	< 0.001
Neutrophils (10^9^/L)	6.4 ± 2.9	6.9 ± 3.3	6.0 ± 2.6	0.077
Hemoglobin (g/L)	112.1 ± 25.9	127.6 ± 21.5	99.2 ± 22.0	< 0.001
C3 (g/L)^g^	1.0 ± 0.4	1.2 ± 0.4	0.8 ± 0.3	< 0.001
C4 (g/L)^g^	0.2 ± 0.2	0.3 ± 0.2	0.2 ± 0.1	< 0.001
IgG (g/L)^g^	8.2 ± 4.5	6.1 ± 2.7	9.8 ± 4.9	< 0.001
CD19^+^ B cells (/μL)^h^	151.5 (73.0, 281.0)	210.5 (107.8, 399.2)	108.0 (54.5, 178.8)	< 0.001
CD4^+^ T cells (/μL)^h^	543.0 (287.5, 887.0)	783.5 (465.2, 1007.0)	378.0 (222.0, 668.0)	< 0.001

Categorical variables are expressed as percentage; continuous variables as mean ± standard deviation or medians (interquartile range).

PN, primary nephropathy; SN, secondary nephropathy; BMI, body mass index; LN, lupus nephritis; AAV, anti-neutrophil cytoplasmic antibody (ANCA) associated vasculitis; MN, membranous nephropathy; MCD, minimal change disease; FSGS, focal segmental glomerulosclerosis; SI, severe infection; MMF, mycophenolate mofetil; CsA, cyclosporine A; CYC, cyclophosphamide; LEF, leflunomide; TAC, tacrolimus; GCs, glucocorticoids; RTX, rituximab; eGFR, estimated glomerular filtration rate; ALB, serum albumin; UTP, total 24-hour urinary protein; WBC, white blood cell; C3, complement 3; C4, complement 4; IgG, immunoglobulin G.

a Complement-mediated thrombotic microangiopathy n = 5, Anti-glomerular basement membrane disease n = 3, Cryoglobulinemia-associated nephropathy n = 2, IgA vasculitis with nephritis n = 2 and IgG4-related disease n = 1.

b Any severe infection within 3 months prior to RTX treatment.

c Cumulative dose of glucocorticoids in the last 3 months prior to RTX treatment.

d Dose of RTX = 375mg/m^2^/week×4 infusions.

e Dose of RTX < 375mg/m^2^/week×4 infusions.

f Dose of RTX < 500mg/person.

g Data missing for 6 patients.

h Data missing for 11 patients.

### 3.2 Baseline characteristics

Among the patients, 30.1% initially received RTX-based treatment regimens, while the remaining cases were relapsed or refractory. In the PN group, 75% received the standard dose of RTX, while in the SN group, a higher percentage (67.2%) received a reduced or low-dose RTX regimen. The cumulative dose of RTX in the SN group was significantly lower than that in the PN group, with 1.5 (0.9, 2.1) g versus 2.4 (2.0, 2.6) g, p < 0.001. Prior to RTX treatment, 78.9% of patients had received various doses of GCs, and 65.9% had previously received other immunosuppressants. After the first dose of RTX, 88.6% of patients were treated with a combination of GCs, and 52% received other immunosuppressants. Cyclosporine A (CsA) was the most commonly used immunosuppressant in the PN group, while mycophenolate mofetil (MMF) was more prevalent in the SN group (Detailed data are shown in **Table 2**). Additional information on the immunosuppressive regimens for different diseases in each group prior to and following RTX initiation can be found in **Supplementary Table S1**. The application of prophylactic anti-infective drugs received by patients in the SN and PN groups is detailed in **Table 2**.

**Table 2:** Rituximab regimen and concomitant therapy in 123 patients.

Characteristics	Total (n = 123)	PN (n = 56)	SN (n = 67)	*p* Value
RTX indication, n (%)				0.739
Relapse/refractory disease	86 (69.9)	40 (71.4)	46 (68.7)	
Initial treated disease	37 (30.1)	16 (28.6)	21 (31.3)	
RTX induction protocol, n (%)				< 0.001
Standard dose^d^	64 (52.0)	42 (75)	22 (32.8)	
Reduced dose^e^	43 (35.0)	11 (19.6)	32 (47.8)	
Low dose^f^	16 (13.0)	3 (5.4)	13 (19.4)	
RTX maintenance, n (%)	64 (52.0)	33 (58.9)	31 (46.3)	0.162
Cumulative dose of RTX (g)	2.0 (1.2, 2.5)	2.4 (2.0, 2.6)	1.5 (0.9, 2.1)	< 0.001
RTX duration (months)	10.7 ± 28.5	10.7 ± 11.8	10.7 ± 37.2	0.997
Concomitant treatment, n (%) immunosuppressant	58 (47.2)	31 (35.7)	27 (40.3)	0.042
CYC	7 (5.7)	2 (3.6)	5 (7.5)	0.035
CsA	22 (17.9)	18 (32.1)	4 (5.9)	< 0.001
MMF	14 (11.4)	2 (3.6)	12 (17.9)	0.013
Tacrolimus	10 (8.1)	9 (16.1)	1 (1.47)	< 0.001
Belimumab	7 (5.7)	0 (0)	7 (10.4)	0.016
AZA	2 (1.6)	0 (0)	2 (2.9)	0.5
GCs	109 (88.6)	46 (82.1)	63 (94)	0.039
Prophylactics, n (%)				
TMP-SMX	56 (45.5)	14 (25)	42 (62.7)	< 0.001
Ganciclovir	18 (14.6)	3 (5.4)	15 (22.4)	0.008
Isoniazid	12 (9.8)	5 (8.9)	7 (10.4)	0.777
Entecavir	3 (2.4)	0 (0)	3 (4.5)	0.25
Follow-up time (months)	19.7 ± 14.6	20.8 ± 13.0	18.8 ± 15.9	0.451

Categorical variables are expressed as percentage; continuous variables as mean ± standard deviation or medians (interquartile range).

RTX, rituximab; CYC, cyclophosphamide; CsA, cyclosporine A; MMF, mycophenolate mofetil; AZA, azathioprine; GCs, glucocorticoids; TMP-SMX, trimethoprim-sulfamethoxazole.

### 3.3 Incidence and main features of SIs

Overall, 32 of 123 patients (26%) experienced 39 cases of SIs during a median follow-up period of 19.7 ± 14.6 months, resulting in an incidence rate of 18.9/100 patient-years. The incidence of SIs was significantly higher in patients in the SN group compared to the PN group (n=27 [40.29%] vs. n=5 [8.9%], P<0.001). Among patients in the SN group, the infection rate was 40.5% (15/37) in patients with LN, 35.3% (6/17) in patients with AAV, and 46.2% (6/13) in patients with other diagnoses. Five patients experienced two infectious episodes, while one patient had three. Four patients had SIs of grades 4 and 5 (n = 2, respectively; all in the SN group) according to the CTCAE v5.0 criteria.

**Figure 2:**
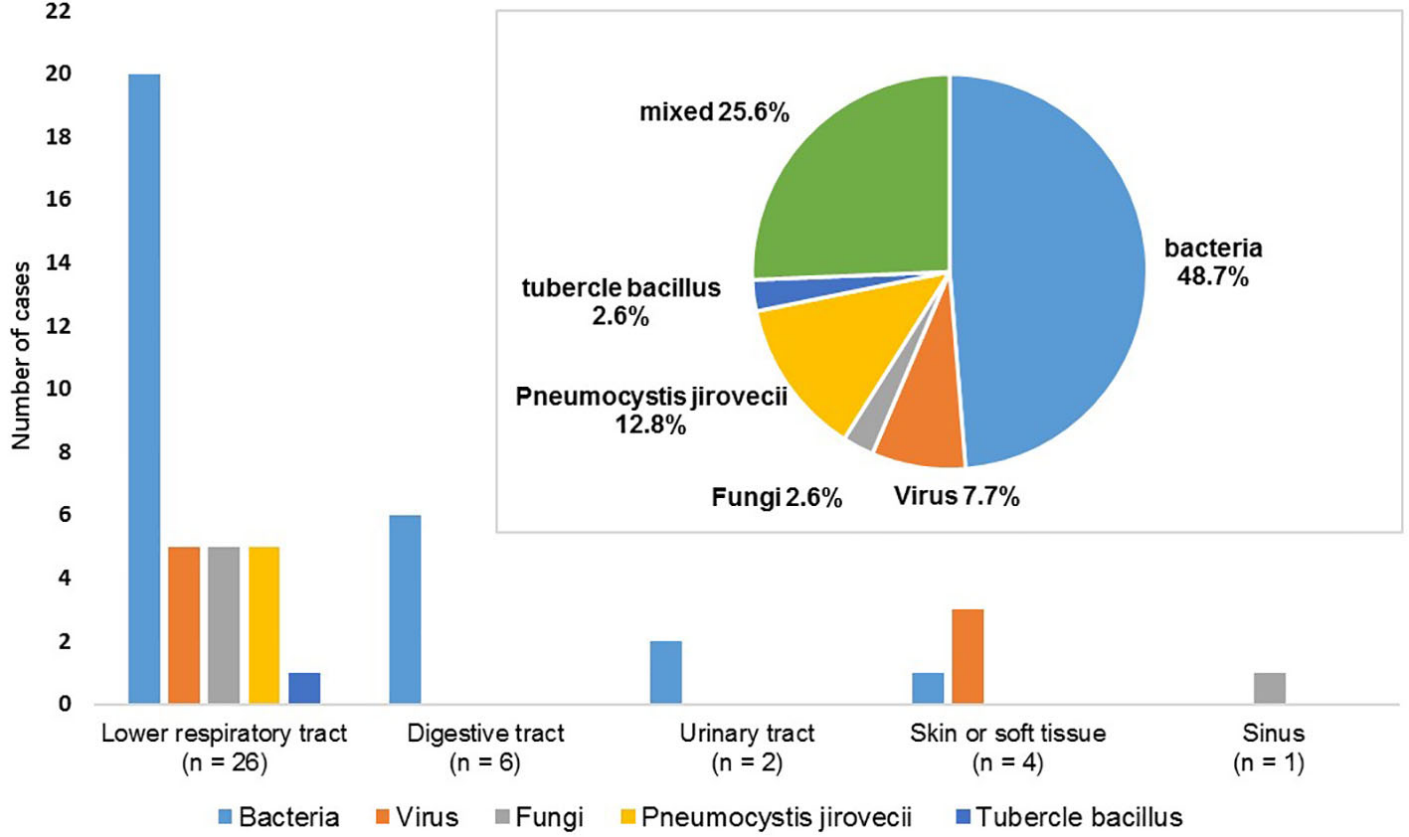
Distribution of 39 cases of severe infections according to the infection sites and pathogens.

The spectrum of the 39 cases of SIs is depicted in **Figure 2**. The lower respiratory tract was the most frequently infected site (66.7%) followed by the gastrointestinal tract (15.4%). Bacteria were the most common pathogenic microorganisms responsible for infections, accounting for 74.4% of cases. Our study also identified 9 opportunistic infections (7.3%), including 5 cases of Pneumocystis carinii pneumonia, one case of pulmonary aspergillosis, 2 cases of herpes zoster, and one case of pulmonary tuberculosis. Additionally, there were 10 cases (8.1%) of mixed infections with more than two pathogens, including bacteria, viruses (cytomegalovirus in 4 cases, SARS-Cov2 in 1 case), and fungi (Aspergillus in 1 case, Pneumocystis carinii in 1 case, Candida in 4 cases).

The study period from 2017 to the end of 2022 partially coincided with the COVID-19 pandemic. During the peak of the pandemic, no new patients were started on RTX treatment in our center, and those already on RTX treatment had fewer follow-up visits to the hospital, which may have resulted in some missing follow-up data. However, this did not greatly affect the collection of data on endpoint events, as information on serious infectious complications was readily available through telephone follow-up. Throughout the study period, five patients reported infection with COVID-19 (confirmed by SARS-Cov2 antigen or nucleic acid testing), with the majority having mild or no symptoms. Only one case of grade 3 serious infection occurred. The patient was a 27-year-old female with lupus nephritis who developed pneumonia with bacterial and SARS-Cov2 co-infection 8 month after RTX treatment. She was hospitalized for 19 days and then discharged. This isolated case suggests that the COVID-19 pandemic had minimal influence on our study.

Among patients who developed SIs, the average IgG level and CD19+ B-cell counts at the time of infection were 6.5 (5.1–8.1) g/L and 1 (0–2)/μL, respectively (refer to **Supplementary Table S2** for more details). B-cell counts greater than 5/µL were observed only in two patients (7/µL and 10/µL, respectively). Additionally, the median daily dose of GCs (in terms of prednisone or its equivalents) was 23.8 (11.9–40) mg. The median interval between the first RTX infusion and first infectious episode was 61 (36, 191.5) days. Thirty-two infectious episodes were noted in 27 patients (21.9%) within 1 year after RTX application, resulting in an infection rate of 19.3/100 patient-years. A total of 5 patients experienced their first SIs 1 year after receiving the first dose of RTX. Among them, 3 (2.43%) patients experienced late infection, indicating that their first infection occurred one year after receiving the last dose of RTX. The detailed clinical information of these 5 patients is provided in **Supplementary Table S3**.

### 3.4 Association between nephropathy subgroups and SIs

**Figure 3:**
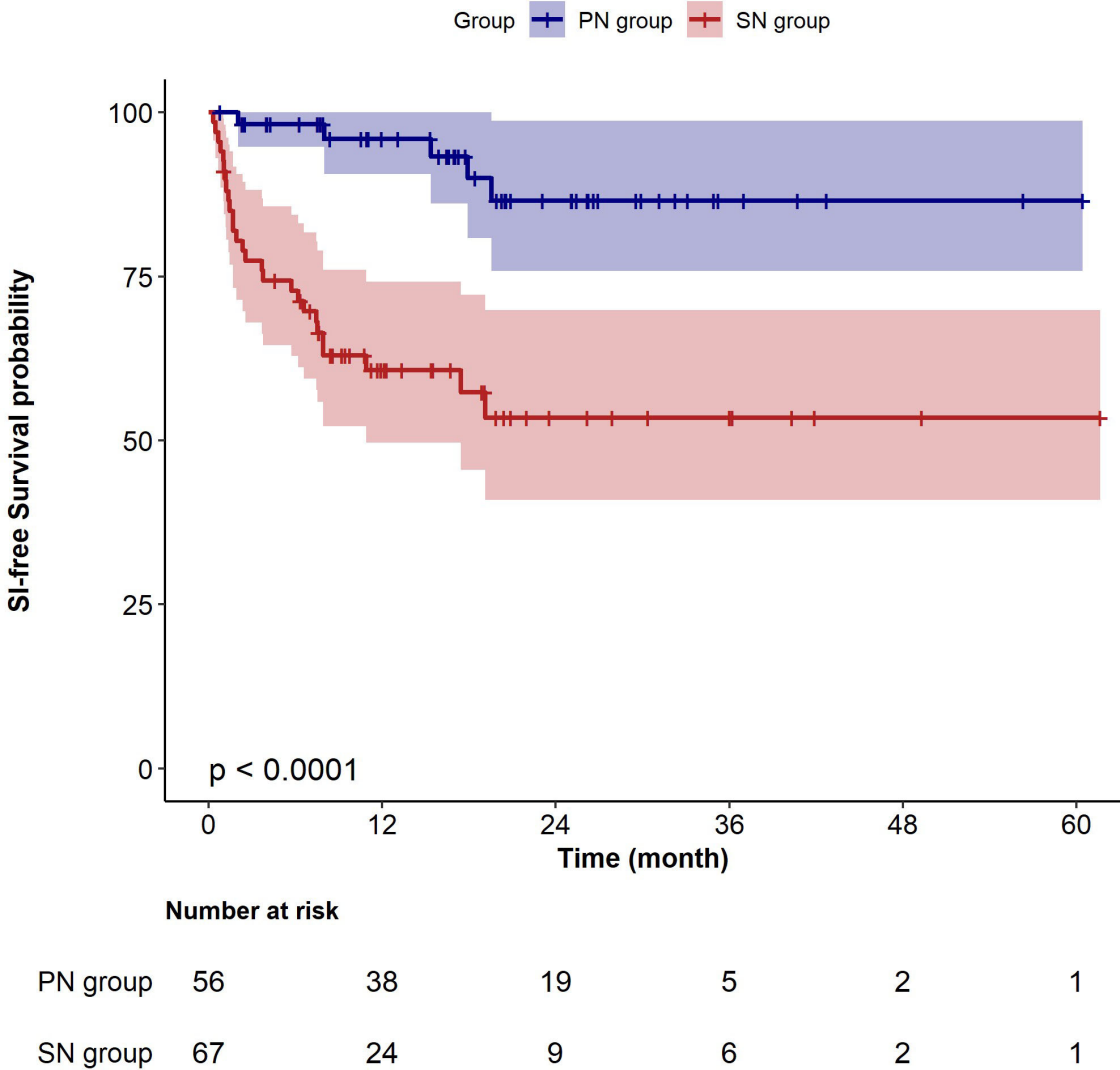
Kaplan–Meier survival curves for sever infection-free survival in patients with primary nephropathy and secondary nephropathy. PN, primary nephropathy; SN, secondary nephropathy.

In the extended multivariable Cox models, the inclusion of covariates was gradually expanded. Model III included a comprehensive set of covariates, including gender, age, BMI, disease duration, baseline eGFR, 24-hour urinary protein level, Lymphocyte count, IgG level, recent history of SIs, dialysis, cumulative dose of GCs pre- and post-RTX treatment, Previous and co-use of immunosuppressant, co-use of cyclosporine A, mycophenolate mofetil, cyclophosphamide, and belimumab. The results consistently showed significant HRs for SN versus PN in all six models (HR range 4.14–6.05, P < 0.05 for all, **Table 3**). Kaplan–Meier curves showed that patients with SN have a higher risk of developing SIs compared to those with PN (Log-rank test: P < 0.0001, **Figure 3**).

**Table 3:** Multivariable Cox regression analyses on association between nephropathy subgroups and severe infections following rituximab treatment.

Group	total	incidence	Basic model		Model I		Model II		Model III	
	n	n (%)	HR (95%CI)	*p*	HR (95%CI)	*p*	HR (95%CI)	*p*	HR (95%CI)	*p*
PN	56	5 (8.9)	1 (Ref)		1 (Ref)		1 (Ref)		1 (Ref)	
SN	67	27 (40.3)	4.14 (1.46~11.76)	<0.001	4.68 (1.10~20.67)	0.042	6.05(1.32~27.79)	0.021	5.86 (1.05~32.63)	0.044

Basic model: adjusted for gender, age, and BMI.

Model I: adjusted for basic model plus baseline eGFR, total 24-hour urinary protein, lymphocyte count, IgG levels, any recent history of severe infection, previously use of cyclophosphamide, cumulative dose of glucocorticoids 3 months prior to rituximab, and cumulative dose of rituximab.

Model II: adjusted for model I plus disease duration, co-use of glucocorticoids.

Model III: adjusted for model II plus co-use of immunosuppressant, co-use of cyclosporine A, co-use of mycophenolate mofetil, co-use of cyclophosphamide, and co-use of belimumab.

### 3.5 Sensitivity analyses

The results of multiple propensity score analyses were consistent with those from univariable and multivariable COX regression models (**Table 4****).** After PSM, the variables including age, BMI, disease duration, RTX treatment indication (relapsed or refractory cases/initial treated cases), cumulative dose of RTX, co-use of GS, co-use of immunosuppressant were found to be similar or “balance” (SMD value <0.1) between the PN and SN groups in the matched cohort, as shown in **Supplementary Table S4**. The results of the subgroup analyses suggest that the relationship between nephropathy subgroups and SIs remained significant even after excluding individuals with missing baseline IgG levels or who were on hemodialysis before receiving RTX treatment. (**Supplementary Tables S5**, **S6**). The cohort had an E-value of 5.99, indicating that the observed RR can only be completely attributed to unmeasured confounding when there is a simultaneous association between the presence of unmeasured confounders and both the nephropathy subgroups and the SIs, with an RR of at least 5.99, and when known confounders are accounted for.

**Table 4:** Associations between types of kidney disease and severe infections after rituximab administration in the propensity score analyses.

Method	OR (95%CI)	P value
Crude analysis	6 (2.3~15.64)	<0.001
Multivariable adjusted^a^	7.4 (2.02~27.01)	0.002
Propensity score analyses^a^		
Adjusted for propensity score	4.49 (1.34~15.05)	0.015
With matching	10.11 (1.26~81.09)	0.029
With IPW	4.84 (1.45~16.16)	0.005
With SMRW	9.85 (1.77~54.77)	0.006
With PA	6.01 (0.91~39.69)	0.027
With Ow	6.3 (0.62~64.52)	0.015

IPW, the inverse probability weighting; SMRW, standardized mortality ratio weighting; PA, pairwise algorithmic; OW, overlap weight.

a Variables included in the model: sex, age, BMI, disease duration, RTX treatment indication (relapse or refractory/Initial treated disease), cumulative dose of RTX, co-use of GS, co-use of immunosuppressant, and baseline IgG levels.

### 3.6 Other risk factors for SIs

In addition to the type of kidney disease (SN vs. PN), multivariable Cox regression analysis revealed advanced age (HR: 1.03; 95% CI: 1.01–1.06; P = 0.023) and recent history of SIs (HR: 5.68; 95% CI: 2.2–14.66; P < 0.001) as contributing factors for SIs. Baseline IgG levels were negatively associated with the risk of SIs (HR: 0.75; 95% CI: 0.64–0.89; P < 0.001), with a 25% decrease in the risk of SIs for every 1 g/L increase in baseline IgG levels. This implies an increased risk of SIs in patients with reduced baseline IgG levels. However, gender, BMI, disease duration, eGFR at baseline, urine protein levels, lymphocyte count, previously use or co-use of GCs or immunosuppressant were not significant factors (**Supplementary Table S7**).

## 4 Discussion

In our study, the incidence of SIs in patients with kidney disease treated with RTX was 18.9/100 patient-years. While most infections occurred within the first year of RTX treatment, there were cases of late-onset infections. Bacterial infections were the most frequently observed, although opportunistic and mixed infections were not uncommon. We identified a strong association between SIs and the type of kidney disease. Patients with SN were at a significantly higher risk for SIs compared with those with PN. Particularly, elderly patients, those with low basal IgG levels, or patients with recent severe infections were identified as high-risk groups for developing SIs following RTX treatment. Given variations in baseline data between PN and SN groups, as well as the numerous factors influencing infection occurrence such as the use of GCs or immunosuppressive agents, and patients’ basal immune status, we constructed multiple COX regression models incorporating different variables. Additionally, PS analysis was employed to partially mitigate selection bias. To further support our findings, we calculated the E-value to evaluate unmeasured confounding.

Several studies have reported that the peak period of infections after RTX treatment was between 3–6 months, with the respiratory system being the most frequent site of infections and bacteria being the primary cause; the results of our study were comparable to these findings. However, in our study, a notable proportion of patients (5.69%) experienced their first SIs 1 year after the initial infusion of RTX. Additionally, we observed that B-cell counts remained depleted in most patients at the time of infection, indicating a prolonged effect of RTX in patients with kidney disease. A similar situation was observed in a study by Trivin et al., where two patients developed mycobacterial infections 24 and 28 months after the last RTX infusion (12). Nevertheless, we did observe a small number of patients showing signs of B cell recovery. It is still uncertain whether infections occurring after B-cell recovery are related to RTX, as there are currently no established criteria for determining this association. Hence, we referred to the infections observed in our study as “infections following RTX treatment” rather than “RTX-related infections”. Additionally, our observations indicate that approximately 15% of patients developed opportunistic infections, accounting for up to 48.7% of SI events. While there is limited data on the incidence of SIs caused by viruses, Mycobacterium tuberculosis, fungi, or Pneumocystis carinii after RTX treatment, it is important to note that cases have been reported and should not be overlooked (8, 10, 12, 20–23). Therefore, nephrologists should take a more comprehensive approach in preventing, monitoring, and evaluating infections after RTX treatment.

The incidence of SIs in patients with autoimmune kidney disease treated with RTX varies considerably in the literatures. However, the incidence is generally low in patients with PN. For example, in the MENTOR study (randomized controlled trial), only 6% of patients with MN treated with RTX developed SIs during the 2-year follow-up period (1). Meanwhile, some cohort and case series studies have reported 0% incidence among patients with MN (2, 3). Cohort studies on patients with refractory nephrotic syndrome, MCD, or FSGS have shown that the incidence of SIs after RTX treatment ranges from 0% to 4.9% (4–6). In contrast, the incidence of SI was significantly higher in patients with SN who received RTX. The LUNAR study (randomized controlled trial) reported that SIs occurred in 19.2% of patients with LN treated with RTX (8). A meta-analysis found that the incidence of SIs after RTX administration in LN was 15.8% (7). Furthermore, a multi-center retrospective cohort study reported an SI prevalence of 26.06/100 patient-years following RTX therapy in patients with AAV (22); this finding is consistent with the results of two single-center cohort studies, which reported SI incidence rates of 20.9 and 28/100 patient-years, respectively (10, 11). The cited studies suggest a possible relationship between the type of kidney disease and the occurrence of SIs. However, it is important to note that this association has not yet been confirmed through research.

To our knowledge, only two studies have been conducted specifically on the incidence of SIs after RTX application in autoimmune nephropathy (12, 13). The populations in both studies were from European countries. Trivin et al. identified diabetes mellitus, cumulative dose of RTX, and concomitant use of azathioprine, while Odler et al. reported that body mass index and baseline creatinine levels were significantly linked to SIs. The risk factors screened in our study differed from the results of the two previous studies. This discrepancy may be attributed to several reasons. First, our research focused on Asian populations. Second, the distribution of kidney diseases in our study population differed from that of the previous studies. Third, we considered all serious infections (SIs) that occurred during the follow-up period after RTX administration, even if they occurred after 1 year of treatment. Additionally, our multivariable analyses incorporated extra variables such as cumulative GC dose before RTX treatment, history of non-RTX-related infections, and particularly, we investigated the impact of nephropathy type (SN versus PN) on the risk of SIs. Our study found that several other risk factors, including advanced age, low basal IgG levels, and a recent history of non-RTX-related SIs were associated with the development of SIs in addition to the type of nephropathy. Research findings have widely recognized the increased risk of infection with age (10, 12, 22). Our study suggests that low baseline IgG levels increase the risk of infection after RTX therapy, a finding that, to our knowledge, has not been reported in previous studies involving patients with kidney disease. Several previous studies conducted on patients with B-cell lymphoma and patients with autoimmune disease indicate that low baseline IgG levels are associated with an increased risk of hypogammaglobulinemia after RTX treatment (24–26). Furthermore, a study by Manuel Alfredo Podestà et al. revealed that hypogammaglobulinemia was prevalent six months after induction in AAV patients treated with rituximab, with lower IgG levels being linked to severe infections (27). The recent history of a non-RTX-related SIs indicate that patients are at higher risk for infections due to factors such as inherent immunity, underlying diseases, and previous immunosuppressive therapy. We included this screening variable as it is easily accessible in the medical history, and our analysis revealed its usefulness in assessing infection risk.

Our COX regression analyses revealed a significant negative association between the cumulative dose of RTX and the risk of SIs, as shown in in **Supplementary Table S7**. This association stemmed from the disparity in RTX dosage between the two patient groups with nephropathy. The baseline data from our study (**Table 2**) indicated that a majority of patients with SN received reduced or low-dose RTX regimens, resulting in a significantly lower cumulative dose compared to patients in the PN group [1.5 (0.9, 2.1) Vs. 2.4 (2.0, 2.6), p<0.001]. Even after balancing the cumulative RTX dose through propensity score matching, the results continued to demonstrate a significant association between the type of nephropathy and SIs (**Table 4**, **Supplementary Table S4**). Essentially, individuals in the SN group exhibited a higher susceptibility to infections compared to those in the PN group, despite receiving lower RTX doses, leading to the illusion that those with lower cumulative RTX doses faced a higher infection risk. This further supports the notion that patients with SN are at a heightened risk of infection, with nephropathy type being independently linked to SIs following RTX treatment. Research on the relationship between cumulative doses of RTX and the risk of SIs is limited and has yielded varying results. Filanovsky et al. discovered that patients with lymphoma who received more than 8 doses of RTX experienced a threefold increase in SIs (28). In the study by Trivin et al, patients with autoimmune nephropathy were administered a mean total dose of 2800 (2000–3562) mg of RTX, revealing a slight but statistically significant association between the total dose of RTX and a composite endpoint of severe infection or death (OR: 1.0005; 95% CI: 1.0001–1.0009; P = 0.01) (12). On the other hand, Li et al. reported a high incidence of serious infections (37%, equivalent to 20.9/100 person-years) in an AAV population (96.3% with renal involvement) treated with a low dose of RTX (mean cumulative dose of 1.27 g) in an observational study (10). Perhaps there is not a linear correlation between the cumulative dose of RTX and the risk of SIs, which needs to be confirmed by further studies.

Infection is a common adverse effect of immunosuppressants. However, our study did not find a significant association between the previous use or concurrent use of GCs or immunosuppressants and the risk of SIs. Several reasons may explain this. First, it is important to note that the inclusion of other influential factors, such as the type of nephropathy (SN vs PN), advanced age, previous history of SIs, and low baseline IgG level, may have the potential to obscure the impact of immunosuppression on outcomes. Second, the comparability between groups was hindered by the presence of various disease types and different immunosuppressive regimens in the study population. Third, the limited sample size for each immunosuppressive agent also affected the statistical power of the study.

Additionally, our study had other limitations. First, this study is retrospective, so it is important to acknowledge the presence of selection bias and potential confounders. However, we conducted sensitivity analyses to mitigate possible bias and confounding to some extent. Second, there were missing data at baseline and during follow-up, including IgG levels and CD19+ B-cell counts. However, a subgroup analysis was conducted by repeating the study after excluding patients with missing values, which yielded stable results. Third, this was a single-center study with a limited sample size, and the majority of the study population consisted of patients with MN and LN. In the future, conducting multi-center prospective observational studies with larger sample sizes or randomized controlled trials will be necessary to obtain more accurate results.

## 5 Conclusion

Our study revealed a significant prevalence of SIs in patients with autoimmune kidney disease following RTX treatment. However, the risk of SIs varied significantly between primary and secondary nephropathy. Patients with secondary kidney disease exhibited a high vulnerability to infections, whereas those with primary kidney disease were comparatively safer. Several key factors, such as advanced age, history of non-RTX-associated SIs, and low basal IgG levels, may assist in identifying individuals at high risk for RTX-associated SIs.

## Data availability statement

The original contributions presented in the study are included in the article/**Supplementary Material**. Further inquiries can be directed to the corresponding author.

## Ethics statement

Informed consent was not obtained from the participants, as it was a noninterventional retrospective data analysis of real-life data collected on patients’ regular visits. This research was conducted in accordance with the Declaration of Helsinki and was approved by the clinical research ethics committee of the Peking University International Hospital (Ethics No. 2022-KY-0028–01).

## Author contributions

JX: Writing – review & editing, Software, Investigation, Formal analysis, Data curation, Conceptualization, Writing – original draft. YD: Writing – review & editing, Formal analysis, Data curation, Conceptualization. ZQ: Writing – review & editing, Validation, Methodology, Investigation, Formal analysis, Data curation, Conceptualization. FY: Writing – review & editing, Supervision, Formal analysis, Conceptualization.
