# Supplementary material for: Differences in risk of serious infections between patients with secondary versus primary nephropathy following rituximab treatment: a retrospective cohort study
Source: Front Immunol. 2024 Jun 11;15:1390997. doi: 10.3389/fimmu.2024.1390997 (PMC11196396; doi:10.3389/fimmu.2024.1390997)
Supplement: Supplementary file 1 [file DataSheet_1.pdf]

SUPPLEMENTARY MATERIAL

Supplementary Table S1. Immunosuppressive regimens before and after RTX administration and incidence of severe infections in patients with different types of diseases in SN and PN groups

Variables	Total (n = 123)	SN group			PN group			P value
		LN (n = 37)	AAV (n = 17)	Others (n = 13)	MN (n = 42)	MCD (n = 11)	FSGS (n = 3)	
RTX indication, n (%)								< 0.001
Relapse/refractory	86 (69.9)	35 (91.9)	6 (35.3)	5 (38.5)	26 (61.9)	11 (100)	3 (100)	
Initial treated	37 (30.1)	2(5.4)	11 (64.7)	8 (61.5)	16 (38.1)	0 (0)	0 (0)	
RTX regimen								
Total dose (g)	1.9 ± 1.0	1.7 ± 0.8	1.5 ± 1.2	1.2 ± 1.0	2.2 ± 0.7	3.0 ± 1.3	2.3 ± 0.3	< 0.001
Duration (months)	10.7 ± 28.5	15.2 ± 49.1	7.8 ± 13.3	2.0 ± 3.2	9.7 ± 11.3	15.1 ± 13.4	9.3 ± 13.6	0.767
Previously used IS, n (%)	81 (65.9)	35 (94.6)	6 (35.3)	4 (30.8)	24 (57.1)	10 (90.9)	2 (66.7)	< 0.001
CYC	50 (40.7)	31 (83.8)	6 (35.3)	1 (7.7)	10 (23.8)	2 (18.2)	0 (0)	< 0.001
CSA	28 (22.8)	4 (10.8)	0 (0)	0 (0)	17 (40.5)	6 (54.5)	1 (33.3)	< 0.001
MMF	24 (19.5)	16 (43.2)	0 (0)	3 (23.1)	4 (9.5)	1 (9.1)	0 (0)	< 0.001
TAC	14 (11.4)	5 (13.5)	0 (0)	0 (0)	6 (14.3)	2 (18.2)	1 (33.3)	0.197
LEF	17 (13.8)	12 (32.4)	0 (0)	2 (15.4)	2 (4.8)	1 (9.1)	0 (0)	0.004
Previously used GCs, n (%)	97 (78.9)	37 (100)	13 (76.5)	8 (61.5)	25 (59.5)	11 (100)	3 (100)	< 0.001
Co-used IS, n (%)	64 (52.0)	21 (56.8)	7 (41.2)	5 (38.5)	23 (54.8)	7 (63.6)	1 (33.3)	0.019
CYC	7 (5.7)	5 (13.5)	0 (0)	0 (0)	2 (4.8)	0 (0)	0 (0)	0.568
CSA	22 (17.9)	4 (10.8)	0 (0)	0 (0)	14 (33.3)	4 (36.4)	0 (0)	0.003
MMF	14 (11.4)	8 (21.6)	1 (5.9)	3 (23.1)	2 (4.8)	0 (0)	0 (0)	0.085
TAC	9 (7.3)	1 (2.7)	0 (0)	0 (0)	5 (11.9)	2 (18.2)	1 (33.3)	0.048
AZA	2 (1.6)	1 (2.7)	1 (5.9)	0 (0)	0 (0)	0 (0)	0 (0)	0.501
belimumab	7 (5.7)	7 (18.9)	0 (0)	0 (0)	0 (0)	0 (0)	0 (0)	0.007
LEF	3 (2.4)	1 (2.7)	0 (0)	1 (7.7)	0 (0)	1 (9.1)	0 (0)	0.177
Co-used GCs, n (%)	109 (88.6)	35 (94.6)	17 (100)	11 (84.6)	32 (76.2)	11 (100)	3 (100)	0.045
SIs, n (%)	32 (26.0)	15 (40.5)	6 (35.3)	6 (46.2)	3 (7.1)	2 (18.2)	0 (0)	0.003

Abbreviations: SN, secondary nephropathy; PN, primary nephropathy; LN, lupus nephritis; AAV, anti-neutrophil cytoplasmic antibody (ANCA) associated vasculitis; MN, membranous nephropathy; MCD, minimal change disease; FSGS, focal segmental glomerulosclerosis; GCs, glucocorticoids; IS, immunosuppressant; CYC, cyclophosphamide; CsA, cyclosporine A; MMF, mycophenolate mofetil; TAC, tacrolimus; AZA, azathioprine; LEF, leflunomide; SI, severe infection.

Supplementary Table S2. $CD19^+$ B cells counts and IgG levels in patients at baseline and at the time of severe infection

Variables	Total (N = 123)		PN (n = 56)		SN (n = 67)		P value	statistic
	n[a]	value	n[a]	value	n[a]	value		
CD19+ B cells, (/μL)								
Baseline	112	151.5 (73, 281)	54	210.5 (107.8, 399.2)	58	108.0 (54.5, 178.8)	< 0.001	12.207
At the time of SIs	24	1.0 (0.0, 1.2)[b]	5	0.0 (0.0, 1.0)	19	1.0 (0.0, 1.2)	0.677	0.174
IgG level (g/L)								
Baseline	117	8.2 ± 4.5	50	6.1 ± 2.7	67	9.8 ± 4.9	< 0.001	24.3
At the time of SIs	23	7.3 ± 4.2[c]	4	6.3 ± 1.6	19	7.5 ± 4.5	0.627	0.243

Continuous variables are expressed as mean ± standard deviation or medians (interquartile range).

[a] Number of patients for whom data are available.

[b] p value < 0.001 compared to baseline levels.

[c] p value = 0.825 compared to baseline levels.

Supplementary Table S3. Characteristics of 5 patients with first severe infection after 1 year of rituximab treatment

Characteristics	Patient No.				
	1	2	3	4	5
Gender	Male	Female	Male	Male	Female
Age (year)	70	64	70	58	32
Diagnosis	MN	MCD	MN	LN	LN
Disease duration（months）	9	120	31	12	156
Total dose of RTX (g)	1.7	2	2.4	1.2	2
RTX maintenance	Yes	Yes	No	No	No
Concomitant immunosuppressant	Non	Non	Non	Non	MMF, belimumab
First RTX infusion to onset of SI (day)	461	537	587	574	489
Last RTX infusion to onset of SI (day)	71	120	538	443	453
Diagnose of infection	Pneumonia	Sinusitis	Pneumonia	Perianal abscess	Pneumonia
Pathogens	bacteria	Aspergillus	bacteria	bacteria	bacteria，Coronavirus
Dose of GCs at the onset of SI [a] (mg)	0.0	5.0	10.0	17.5	5.0
eGFR at baseline (ml/min/1.73m^2)	14.33	71.5	33.05	16.15	12.35
eGFR after 1 year of treatment(ml/min/1.73m^2)	57.65	66.8	23.9	10.4	38
eGFR at the onset of SI (ml/min/1.73m^2)	39.8	74.0	23.0	8.0	31.0
IgG at the onset of SI (g/L)	8.38	NA	6.51	7.79	NA
CD19+ B cell at the onset of SI (/μL)	0	0	1	1	NA

Abbreviations: MN, membranous nephropathy; MCD, minimal change disease; LN, lupus nephritis; RTX, rituximab; MMF, mycophenolate mofetil; SI, severe infection; GCS, glucocorticoids; eGFR, estimated glomerular filtration rate; IgG, immunoglobulin G; NA, not available.

[a] In terms of prednisone or equivalent.

Supplementary table S4. Characteristics of patients in the PN and SN groups before and after propensity score matching.

	Unmatched cohort			Matched cohort		
Covariate	PN	SN	SMD	PN	SN	SMD
n	56	67		23	23	
Female, n (%)	11 (19.6)	38 (56.7)	0.826	6 (26.1)	8 (34.8)	0.19
Age (year)	52.21 (16.87)	46.60 (19.09)	0.312	47.27 (18.29)	46.95 (19.37)	0.017
BMI (kg/m^2)	25.34 (4.04)	22.99 (2.89)	0.67	24.00 (3.04)	24.22 (3.26)	0.069
Disease duration (months)	28.95 (30.72)	72.04 (91.13)	0.634	36.20 (33.85)	40.57 (45.39)	0.109
Initial treated disease (%)	16 (28.6)	21 (31.3)	0.061	6 (26.1)	6 (26.1)	<0.001
Cumulative dose of RTX (g)	2.39 (0.87)	1.54 (0.96)	0.924	2.27 (0.87)	2.18 (0.98)	0.089
Co-use of GS, n (%)	46 (82.1)	63 (94.0)	0.373	21 (91.3)	21 (91.3)	<0.001
Co-use of IS, n (%)	25 (44.6)	34 (50.7)	0.122	11 (47.8)	11 (47.8)	<0.001
IgG (g/L)	6.29 (2.61)	9.81 (4.86)	0.903	7.85 (2.45)	7.46 (3.58)	0.128

Categorical variables are expressed as number (percentage); continuous variables as mean (standard deviation)

PN, primary nephropathy; SN, secondary nephropathy; SMD, standardized mean difference; BMI, body mass index; RTX, rituximab; GCs, glucocorticoids; IS, immunosuppressant; IgG, immunoglobulin G.

Supplementary Table S5. Sensitive analysis excluding a subset of the population with missing baseline IgG data

group	Total, n	Incidence, n (%)	Crude HR (95%CI)	P Value	Adjusted HR[a] (95%CI)	P Value
PN	50	5 (10)	1(Ref)		1(Ref)	
SN	67	27 (40.3)	5.39 (2.07–14.06)	<0.001	6.33 (1.71–23.43)	0.006

PN, primary nephropathy; SN, secondary nephropathy.

[a] Adjusted for Age, gender, body mass index, baseline eGFR, total 24-hour urinary protein, baseline IgG levels, cumulative dose of glucocorticoids in the last 3 months prior to rituximab treatment, history of any severe infection within 3 months prior to rituximab treatment, cumulative dose of glucocorticoids within 3 months after first dose of rituximab, and co-use of immunosuppressant.

Supplementary Table S6. Sensitive analysis excluding a subset of the population that was on hemodialysis prior to rituximab treatment

group	Total, n	Incidence, n (%)	Crude HR (95%CI)	*P* Value	Adjusted HR[a] (95%CI)	P Value
PN	56	5 (8.9)	1(Ref)		1(Ref)	
SN	51	17 (33.3)	4.81 (1.77–13.11)	0.002	4.91 (1.09–22.02)	0.038

PN, primary nephropathy; SN, secondary nephropathy.

[a] Adjusted for Age, gender, body mass index, baseline eGFR, total 24-hour urinary protein, baseline IgG levels, cumulative dose of glucocorticoids in the last 3 months prior to rituximab treatment, history of any severe infection within 3 months prior to rituximab treatment, cumulative dose of glucocorticoids within 3 months after first dose of rituximab, and co-use of immunosuppressant.

Supplementary Table S7. COX regression analyses on the risk factors for severe infections after the first RTX administration

	Univariable model		Multivariable model	
Variable	HR (95%CI)	P value	HR (95%CI)	P value
SN vs PN	6.00 (2.3–15.63)	< 0.001	5.86 (1.05~32.63)	0.044
Female	3.08 (1.5–6.3)	0.002	2.38 (0.86–6.58)	0.094
Age (year)	1.00 (0.98–1.02)	0.912	1.03 (1.01–1.06)	0.023
BMI (kg/m2)	0.86 (0.76–0.96)	0.008	0.94 (0.81~1.09)	0.392
Disease duration (months)	1.00 (1.00–1.01)	0.021	1.00 (0.99–1.01)	0.274
Diabetes mellitus	0.54 (0.19–1.55)	0.253	…	
Cumulative dose of RTX (g)	0.42 (0.28–0.63)	< 0.001	0.43 (0.24~0.77)	0.004
Recent history of SI [a]	5.87 (2.79–12.31)	< 0.001	5.68 (2.2~14.66)	<0.001
Previous immunosuppressant	2.22 (0.91,5.39)	0.079	…	
CSA	0.53 (0.21,1.39)	0.198	…	
MMF	1.35 (0.60,3.00)	0.466	…	
CYC	2.62 (1.28,5.36)	0.008	0.72 (0.24~2.17)	0.556

CYC (g)	1.07 (0.99,1.16)	0.078	…	
Previous GCs [b] (g)	1.72 (1.38–2.14)	< 0.001	1.26 (0.89~1.79)	0.192
Concomitant GCs [c] (g)	1.42 (1.08–1.89)	0.013	0.6 (0.06~5.68)	0.657
Concomitant immunosuppressant	0.55 (0.27–1.12)	0.101	3.7 (0.79~17.25)	0.095
CSA	0.26 (0.06–1.07)	0.062	1.95 (0.22~17.26)	0.548
MMF	1.42 (0.55–3.7)	0.468	2.31 (0.43~12.32)	0.327
CYC	1.38 (0.48–3.93)	0.552	2.65 (0.27~25.6)	0.400
belimumab	2.34 (0.82–6.68)	0.112	1.88 (0.39~9.06)	0.430
eGFR (mL/min/1.73 m^2)	0.98 (0.97–0.99)	< 0.001	1.01 (0.99–1.023)	0.296
UTP (g/24 hour)	0.96 (0.90 –1.03)	0.316	…	
ALB (g/L)	1.03 (0.98–1.08)	0.292	…	
WBC (10^9/L)	1.00 (0.89–1.11)	0.936	…	
Neutrophils (10^9/L)	1.05 (0.94–1.17)	0.42	…	
Lymphocytes (10^9/L)	0.47 (0.27–0.81)	0.006	0.70 (0.36~1.35)	0.284
IgG (g/L)	0.95 (0.86–1.04)	0.242	0.75 (0.64–0.89)	<0.001

Abbreviations: SN, secondary nephropathy; PN, primary nephropathy; BMI, body mass index; IgG, immunoglobulin G; GCs, glucocorticoids; SI, severe infection; CsA, cyclosporine A; MMF, mycophenolate mofetil; CYC, cyclophosphamide; LEF, leflunomide; eGFR, estimated glomerular filtration rate; UTP, total 24-hour urinary protein; ALB, serum albumin; WBC, white blood cell; IgG, immunoglobulin G.

[a] Cumulative dose of glucocorticoids within 3 months prior to RTX treatment.

[b] Any severe infection within 3 months prior to RTX treatment.

[c] Cumulative dose of glucocorticoids within 3 months prior to rituximab.
